# Psoriasis flare confused with drug allergy: A collaborative effort is required to treat tuberculosis in the setting of severe psoriasis

**DOI:** 10.1016/j.rmcr.2026.102378

**Published:** 2026-01-22

**Authors:** Quratulain Kizilbash, Adriana Vasquez, Barbara Seaworth, Lynn Horvath

**Affiliations:** a The University of Texas at Tyler Health Science Center, 11937 US Highway 271, Tyler, TX, 75708, USA; b Texas Center for Infectious Disease, 2303 Southeast Military Drive, San Antonio, TX, 78223, USA; c South Texas Veterans Health Care System, 7400 Merton Minter Boulevard, San Antonio, TX, 78229, USA

## Abstract

**Background:**

Psoriasis is a chronic autoimmune disorder. Severe psoriasis is treated with systemic immunosuppressive agents. Systemic immunosuppression increases the risk of tuberculosis (TB) disease. Sudden cessation of immunosuppression seems logical in a TB patient but can lead to psoriasis flares. And when a new rash occurs during TB therapy, drug reaction is usually suspected. This can result in untreated TB disease or intermittent therapy, thus increasing the risk for acquired drug resistance.

**Methods:**

We describe two patients who developed TB disease during immunosuppressive therapy for psoriasis. When the immunosuppressive therapy was stopped, due to TB disease, both patients experienced significant worsening of psoriasis. The skin changes were confused with drug reaction to TB medications. Significant treatment interruptions resulted. A single team of TB physicians and one dermatologist, worked in conjunction to formulate a psoriasis and TB treatment plan. The patients were treated with acitretin and cyclosporine systemically along with topical agents to achieve psoriasis control. Then both were sequentially challenged with one TB medication at a time to ensure no drug reaction occurred while monitored at Texas Center for Infectious Disease. Cyclosporine was then tapered off.

**Results:**

Both patients tolerated the alternative psoriasis regimen and TB therapy well. Both demonstrated clinical, bacteriologic, and radiographic improvement.

**Conclusion:**

Treatment of TB disease in patients with severe psoriasis requires a collaborative effort between the TB treatment team and dermatology. A balanced approach, including treatment of both diseases, is necessary to avoid confusion of psoriasis flare versus drug induced skin reactions from TB medications.

## 1 Introduction

Psoriasis is a chronic autoimmune disorder that frequently affects the skin and joints. Psoriasis therapy has traditionally consisted of topical and systemic steroid therapy. However, psoriasis therapy has been revolutionized over the last two decades with development of tumor necrosis factor (TNF)–α–antagonist therapy. TNF–α–antagonist therapy results in dramatic improvement of psoriasis, but the immunosuppressive characteristics result in an increased risk of tuberculosis (TB) disease, as well as other infections [1]. Janus kinase (JAK) inhibitors result in a reduction of a variety of cytokines including TNF–α and are also used to treat a variety of immune mediated diseases including psoriasis [2]. Interleukin (IL) inhibitors including the IL-12/IL-23 inhibitor ustekinumab have emerged as safe and effective options for the treatment of psoriasis and although there is no clear evidence of increased risk of reactivation of TB, routine annual monitoring for TB is still recommended [3,4]. A review of the literature shows an abundance of articles published in the last decade on the topics of psoriasis and TB. However, most of these articles focus on identification and treatment of latent tuberculosis infection (LTBI) prior to initiation of TNF–α–antagonist therapy [[5], [6], [7]]. When TB disease is diagnosed, the recommendation by most experts is to discontinue biologic therapy for psoriasis and initiate TB therapy [8,9]. There is limited information on when to re-introduce TNF–α–antagonist therapy and JAK inhibitors [10].

We describe two patients with severe psoriasis that subsequently developed TB disease. Their treatment courses were complicated by suspected drug reactions, that were ultimately determined to be psoriasis flares. A team of physicians, including a team of infectious diseases physicians and a dermatologist, developed a treatment plan that first targeted the psoriasis and then the TB. Clear skin at the time of rechallenge of TB medications facilitated the provider's ability to identify subsequent drug reactions.

## 2 Case discussion

### 2.1 Patient #1

**Fig. 1:**
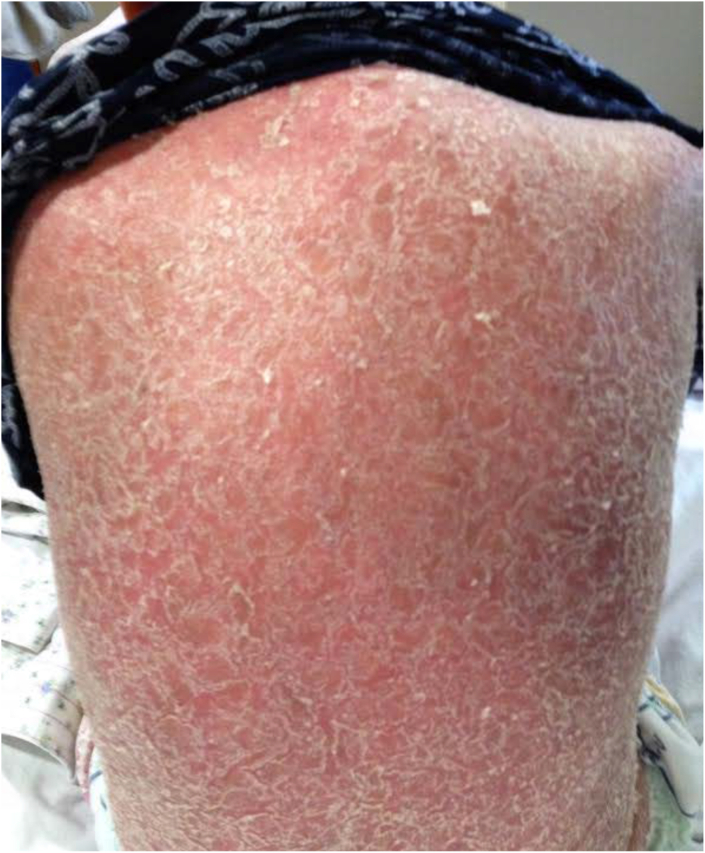
Back with diffuse erythroderma and thick scale

**Fig. 2:**
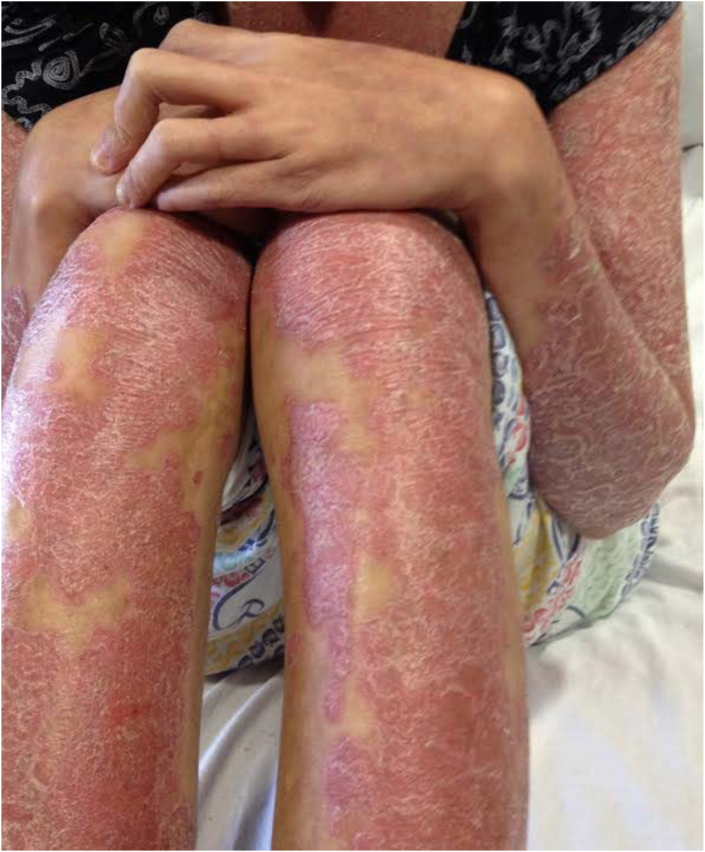
Extremities with diffuse erythroderma and thick scale

Patient is a 42-year-old Asian female with a 13-year history of severe psoriasis complicated by psoriatic arthritis. Her psoriasis had been treated with numerous therapies in the past, including topical agents, phototherapy, acitretin, prednisone, methotrexate, cyclosporine, ustekinumab, adalimumab, etanercept, and infliximab. She was diagnosed with cavitary pulmonary TB disease. At that time, her psoriasis was being successfully treated with etanercept. The patient was started on TB treatment with rifampin, moxifloxacin, pyrazinamide and ethambutol. Isoniazid was not prescribed initially due to concern of possible resistance due to prior isoniazid therapy for 6 months for LTBI. Etanercept was discontinued at the time of TB diagnosis. She was told that she should receive no systemic psoriasis therapy while being treated for TB. Two weeks after starting TB treatment she developed a diffuse rash requiring hospitalization. The rash was thought to be secondary to a TB drug reaction and all TB therapy was stopped. Over the next 6 months she underwent 7 outpatient attempts to restart TB therapy. All resulted in “new” rashes, thought to be drug reactions and led to treatment interruptions. Rifampin and rifabutin were reported as causing the most severe reactions. She was admitted to Texas Center for Infectious Disease (TCID) to be challenged with TB medications in a controlled, hospital setting. Patient had diffuse erythroderma and thick scale at the time of TCID admission (Fig. 1, Fig. 2). It was questioned how any physician could differentiate psoriasis from a drug reaction clinically in this patient. Dermatology evaluated the patient and suggested short term aggressive psoriasis therapy before initiation of TB therapy with acitretin and cyclosporine in conjunction with topical triamcinolone 0.1 % ointment nightly with a sauna suit. Patient's skin dramatically improved and cyclosporine was discontinued two weeks after its initiation. Psoriasis treatment was continued with acitretin, triamcinolone ointment and the sauna suit. TB was treated with sequential introduction of INH, moxifloxacin and linezolid. She then was challenged with rifampin, and she tolerated all drugs without development of rash. Ultimately, she was transitioned to INH and Rifampin therapy and successfully finished a 9-month course of TB therapy.

### 2.2 Patient #2

**Fig. 3:**
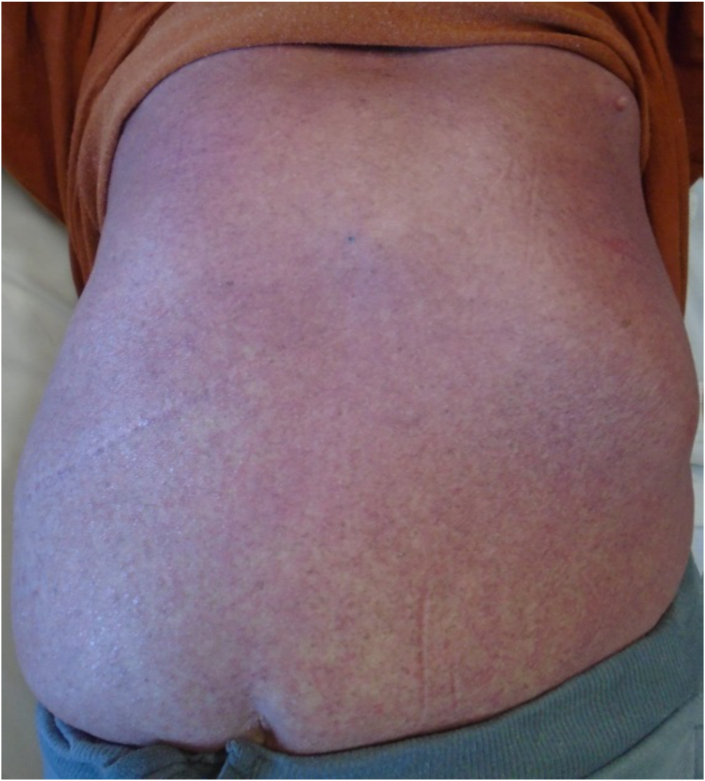
Erythroderma of the back

**Fig. 4:**
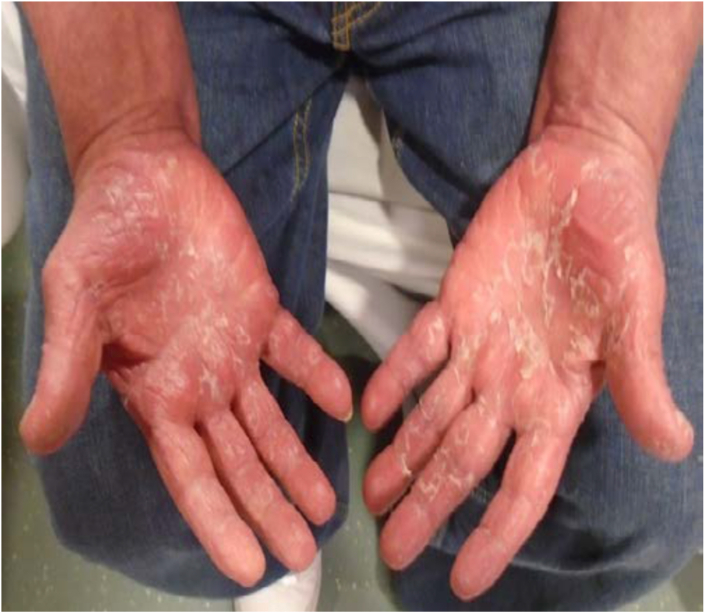
Erythroderma and scaling of the palms

**Fig. 5:**
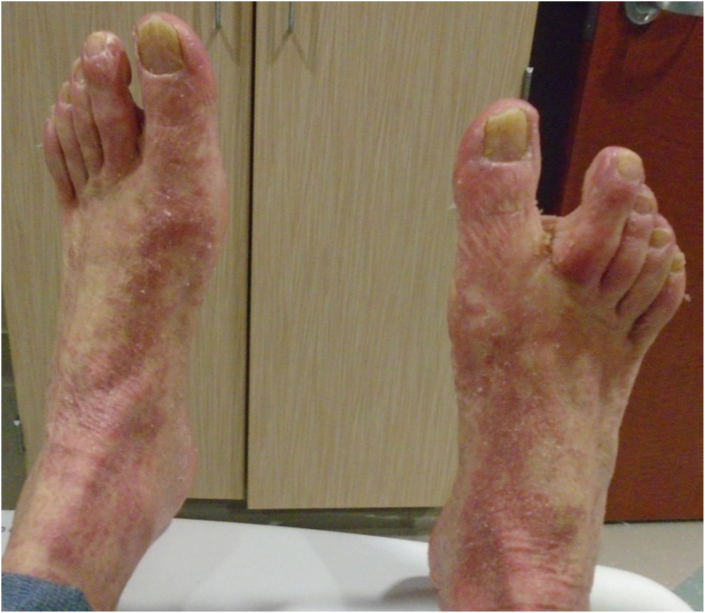
Erythroderma and scaling of the feet with pitting and discoloration of toenails

Patient is a 71-year-old Caucasian male with a 40-year history of psoriasis and diabetes. Treatment for psoriasis included use of tanning beds and ustekinumab which was given 6 months and then again 2 months prior to his presentation to a rheumatologist with fatigue and musculoskeletal discomfort. The psoriasis was well controlled at that time. A screening Chest Xray and subsequent CT chest revealed right hilar lymphadenopathy. He was diagnosed with small cell lung cancer on a right hilar lymph node biopsy and pan-susceptible *Mycobacterium tuberculosis* grew from the same specimen 6 weeks later. He was diagnosed with disseminated TB involving the lungs, liver and spleen and was started on treatment with rifampin, isoniazid, pyrazinamide, and ethambutol and one week later started on chemotherapy for lung cancer with carboplatin and etoposide along with radiation. Two weeks after the initiation of TB therapy, he developed a diffuse, exfoliating rash requiring hospitalization. Drug reaction was considered, but skin biopsy was consistent with a psoriasis flare. One month after re-starting TB therapy, he developed transaminitis (AST 238 and ALT 249), requiring discontinuation of TB therapy. Treatment was changed to a liver friendly regimen with rifabutin, ethambutol and levofloxacin. Due to skin and liver issues, he had numerous treatment interruptions, and had only received 6 weeks of effective TB therapy over a period of 4 months. He was admitted to TCID to be challenged with TB medications in a controlled, hospital setting. Dermatology evaluated the patient and determined he had erythrodermic psoriasis (Fig. 3, Fig. 4, Fig. 5) and recommended systemic psoriasis therapy with acitretin 25mg daily and cyclosporine 100mg three times a day. The patient's pruritus and erythroderma significantly improved. Treatment for TB disease was started 4 days after initiation of psoriasis therapy with an escalating rifabutin dose for two days and then levofloxacin. Patient tolerated these medications well and was discharged on daily levofloxacin and rifabutin for a total of 9 months, to be given by directly observed therapy as an outpatient. Cyclosporine was discontinued two weeks after its initiation. Psoriasis treatment was continued with acitretin, triamcinolone 0.1 % ointment and use of a sauna suit and he was to follow up with dermatology one month after discharge from TCID. He completed radiation and 4 cycles of chemotherapy for lung cancer and was to follow up with oncology for a PET scan and further management.

Two patients were referred to TCID for admission and treatment of TB disease in the setting of severe psoriasis. Both patients were previously unable to tolerate TB therapy due to presumed drug reactions. We utilized a collaborative approach to care with a team of infectious disease physicians and a dermatologist. In both patients, we first aggressively treated the psoriasis. Our goal was to improve the psoriasis before re-challenge with TB medications. In both cases we used a combination of a short term (2 weeks) immunosuppressive agent, cyclosporine, and long-term use of a non-immunosuppressive agent, acitretin, with topical steroid therapy. Both patients demonstrated dramatically improved skin. The patients were then, sequentially challenged with TB medications at TCID while being closely monitored. Both tolerated TB therapy without any skin reaction, their TB disease improved clinically, bacteriologically, and radiographically and they were subsequently discharged to outpatient TB treatment.

Severe psoriasis is often treated with immunosuppressive agents. The diagnosis of TB disease frequently leads to sudden discontinuation of psoriasis therapy. However, an abrupt discontinuation of psoriasis therapy will likely result in a flare of the psoriasis as an immune reconstitution inflammatory syndrome (IRIS) type reaction. Both patients had clear skin prior to the initiation of TB treatment, and they subsequently had a psoriasis flare which was confused with a TB drug reaction. We would recommend continuation of psoriasis treatment if their disease is well controlled. Even in a seriously ill patient with well controlled psoriasis on a TNF–α–antagonist, the best option may be to continue it and start aggressive and adequate TB therapy. If the psoriasis is not controlled at the time of TB diagnosis this may need to be done first if the patient can tolerate waiting several weeks prior to the initiation of TB treatment. If TB treatment is delayed it would be recommended to continue airborne isolation during this time. A collaborative approach, including a TB clinician and a dermatologist, can provide balanced treatment of both TB disease and psoriasis which will allow for TB therapy without interruptions, prevent the development of drug resistance and improve patient satisfaction.

## CRediT authorship contribution statement

**Quratulain Kizilbash:** Writing – original draft, Conceptualization. **Adriana Vasquez:** Writing – review & editing. **Barbara Seaworth:** Writing – review & editing, Conceptualization. **Lynn Horvath:** Writing – review & editing.

## Informed consent

Patients were discharged from the hospital years ago and therefore written consent cannot be obtained at this time however verbal informed consent was provided and poster was presented previously at National TB research meeting without objections from the patients. No patient identifying information has been used for this manuscript.

## Funding sources

This research did not receive any specific grant from funding agencies in the public, commercial, or not-for-profit sectors.

## Declaration of competing interest

The authors declare that they have no known competing financial interests or personal relationships that could have appeared to influence the work reported in this paper.
